# RNA-Seq Analysis Reveals Spatial and Sex Differences in Pectoralis Major Muscle of Broiler Chickens Contributing to Difference in Susceptibility to Wooden Breast Disease

**DOI:** 10.3389/fphys.2019.00764

**Published:** 2019-06-18

**Authors:** Brilynn Brothers, Zhu Zhuo, Michael B. Papah, Behnam Abasht

**Affiliations:** ^1^Department of Biological Sciences, University of Delaware, Newark, DE, United States; ^2^Department of Animal and Food Sciences, University of Delaware, Newark, DE, United States

**Keywords:** wooden breast, broiler, chicken, RNA sequencing, myopathy, gene expression, metabolism

## Abstract

Wooden Breast Disease (WBD) is a novel myopathy affecting the pectoralis major muscle of modern broiler chickens. The etiology of WBD is not currently known, but has been linked to increased feed efficiency, growth rate, and muscle yield in broiler chickens. Differential effect of WBD has been detected between regions of the P. major and between sexes of broilers—male birds and the cranial aspect of the muscle tend to be more severely affected by the disease than females and the caudal aspect. This study aimed to characterize biological differences in the P. major between regions of the muscle and sexes of birds. Samples were taken from the cranial and caudal aspects of P. major muscles of 3-week-old, unaffected male and female birds for RNA sequencing. RNA was extracted and used for preparation of cDNA libraries, which were sequenced by the Delaware Biotechnology Institute (DBI) using HiSeq2500. Sequence reads were aligned to the chicken reference genome with HISAT, and genes were analyzed for differential expression between regions of the breast muscle and sexes of birds using CuffDiff. Functional analysis was performed on differentially expressed genes (DEGs) between sex groups using DAVID and Ingenuity Pathway Analysis (IPA). There were 12 DEGs between cranial and caudal samples, and 260 between male and female birds. Out of the 260 genes differentially expressed between sexes, 189 were upregulated in males. Of this subset, 103 genes (55%) were located on the Z-chromosome. There was increased expression of genes involved in fat metabolism and oxidative stress responses in the cranial region of the P. major muscle, as well as increased expression of fat metabolism, oxidative stress response, antiangiogenesis, and connective tissue proliferation genes in male broilers. These results support the hypothesis that there are biological characteristics in male broilers and the cranial region of the breast muscle that may make them more susceptible to WBD, as well as raising the possibility of a metabolic switch in modern broiler chickens that may be more prominent in males.

## Introduction

Demand for poultry meat has greatly increased over the last 50 years. To meet the still-growing demand for poultry meat, the poultry industry has implemented methods to increase growth rate, feed efficiency, and muscle yield, especially that of the breast muscle, in turkeys and chickens. These improvements in the efficiency of the poultry industry may have inadvertently induced a higher incidence of muscle disorders and abnormalities, such as deep pectoral muscle disease and white striping (Petracci et al., 2013). Myopathies such as these have negative impacts on the poultry industry, as they decrease the quality of chicken breast meat, resulting in the breast being condemned entirely or downgraded for use in manufactured products such as pet food.

Relatively recently, a novel myopathy known as Wooden Breast Disease (WBD) has made an appearance in modern broiler chickens across the world. As described by Sihvo et al. (2014), WBD is characterized by a pale, bulging pectoralis major muscle (P. major) that is palpably firm. Recent histological studies indicate that the earliest signs of WBD appear in the first week post-hatch, when affected birds begin to display localized phlebitis with perivascular lipid infiltration (Papah et al., 2017). Later stages of WBD demonstrate diffuse myodegeneration, inflammatory cell infiltration, fibrosis, necrosis, and lipid infiltration (Papah et al., 2017). Though they may occur independently of each other, WBD is often coexistent with another muscle condition, namely, white striping on the muscle (Sihvo et al., 2014; Mudalal et al., 2015; Dalle Zotte et al., 2017, 2018).

Many aspects of WBD have been characterized at histological and molecular levels, however, its exact etiology is currently unknown. To the best of our knowledge, there has not been any indication of a pathogenic association with the disease, and it is arguable whether or not there is a strong genetic determinism for WBD. However, most agree that the disease is related to the increase in muscle yield, growth rate, and feed efficiency of modern broiler chickens over the past 50 years (Kuttappan et al., 2012; Lorenzi et al., 2014; Sihvo et al., 2014; Russo et al., 2015; Zhou et al., 2015; Abasht et al., 2019), as birds that have not been selectively bred for these economically desirable traits are unaffected by WBD. Additionally, environmental factors impacting growth rate in broilers play major roles in the expression of WBD. In a recent study by Meloche et al. (2018c), reducing feed intake in broilers directly reduced growth rate, and in turn decreased the incidence and severity of WBD, as well as levels of molecular markers for broiler myopathies such as creatine kinase and lactate dehydrogenase. Likewise, reducing digestible lysine, as well as dietary energy and other amino acid densities in feed can reduce the severity of breast muscle myopathies (Meloche et al., 2018a, b). However, birds with reduced nutrient treatments in these studies typically had decreases live performance traits such as body and P. major weight (Meloche et al., 2018a, b). It has also been reported that earlier hatch times and increased temperature during incubation day 14–18 result in fewer and less severe P. major myopathies in broiler chickens (Clark et al., 2017). However, it can not be disregarded that birds undergoing the increased incubation temperature treatment also had lower body weights and P. major weights than control birds (Clark et al., 2017).

A previous study from our laboratory on differential gene expression between WBD-affected and unaffected tissue revealed altered biological pathways correlated with the disease. Mutryn et al. (2015) found over 1500 differentially expressed genes (DEGs) between birds affected and unaffected by WBD, suggesting hypoxia, oxidative stress, connective tissue disorders, and cellular repair mechanisms in WBD-affected tissues. A study by Abasht et al. (2016) on the WBD metabolic profile demonstrates evidence of decreased glucose metabolism through glycolysis. Additional studies on gene expression show evidence of myodegeneration, inflammation, lipid infiltration, and fibrosis (Zambonelli et al., 2016; Papah et al., 2018) in WBD-affected tissues.

In most WBD-affected samples, the cranial aspect of the pectoralis major muscle appears to be more severely affected than the caudal, as it tends to be firmer, thicker, and display a higher degree of white striations than the caudal aspect (Bailey et al., 2015; Clark and Velleman, 2016; Papah et al., 2017). Additionally, it has been observed that male modern broiler chickens have a higher incidence rate of WBD than females, and typically are more severely affected by the disease (Trocino et al., 2015). Considering the correlation of WBD with increased body and breast muscle mass and growth rates, it could be assumed that male birds and the cranial aspect of the pectoralis major muscle are more susceptible to WBD because they grow faster and larger than female birds and the caudal aspect of the muscle. It is a fact that male broiler chickens tend to have higher pectoralis major and overall body masses than females, as well as typically growing at a faster rate (Scheuermann et al., 2003; Tatara et al., 2012; Trocino et al., 2015). Therefore, the key to understanding increased WBD incidence and severity in male broilers and the cranial region of the muscle is understanding the biological differences between sexes of birds and regions of the pectoralis major that are associated with higher growth rates and body size.

This study aims to provide answers for a few questions: Why is the cranial aspect of the pectoralis major more affected by WBD than the caudal, and why are males more affected than females? Additionally, why does the breast muscle grow larger and faster in male birds than females, and why does the cranial aspect grow faster and become thicker than the caudal aspect? This study utilizes RNA sequencing techniques to look at differential gene expression between cranial and caudal sections of the pectoralis major, as well as between male and female birds in search of differing biological pathways that may lead to differing susceptibility to WBD.

## Materials and Methods

### Chickens and Sample Collection

A total of 171 Cobb500 broiler chickens of the same egg age and parent flock were hatched at the University of Delaware poultry farm and placed on the floor with wood shavings in chicken houses with automatic ventilation and a light cycle consisting of 1 h of darkness followed by 23 h of light per day. Houses were kept initially at 95°F and lowered by 5°F each week until they reached 70°F. Birds were fed standard commercial starter and grower diets and were allowed free access to feed and water. Bird houses were kept below a stocking density of 30 kg/m^2^ throughout the entirety of the experiment in accordance with the Animal Care and Use Handbook 2014 of the College of Agriculture and Natural Resources, University of Delaware. The animal protocols were reviewed and approved by the University of Delaware’s Institutional Animal Care and Use Committee.

Birds were weighed each week from the day they hatched until they were humanely euthanized by cervical dislocation at day 7, 14, 21, or 56 of age. Birds euthanized at days 7, 14, and 21 had their sexes determined by identification of gonads during necropsy, as secondary sex characteristics are unnoticeable until around 4 weeks of age. For birds euthanized at day 56, their sexes were determined by observation of secondary sex characteristics.

At 3 weeks of age, 24 birds were randomly selected and euthanized by cervical dislocation followed by necropsy and sample collection for histologic examination and RNA-sequencing. Tissue samples were taken from the cranial and caudal aspects of the P. major muscle of all 24 birds. Samples were approximately 2 g in mass and were harvested along the longitudinal axis of the muscle fibers. To ensure that the same muscle fibers were used for microscopy and RNA-seq, each sample was split into two at the middle perpendicular to muscle fibers, with one portion immediately immersed into 10% neutral buffered formalin for histology, while the other was flash frozen in liquid nitrogen before transfer to -80°C for RNA-seq. The muscle samples were taken from the same regions for all experimental chickens.

### Selection and Identification of Samples for RNA Sequencing

**FIGURE 1:**
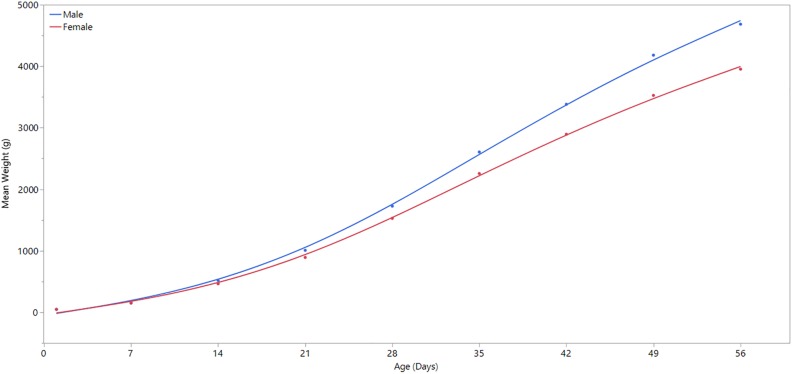
Comparison of weekly body weight by sex. Day 1 refers to the day of hatch. Male broilers have significantly higher average body weights than females at ages 7, 14, 21, 28, 35, 42, 49, and 56 days (*P* ≤ 0.0001).

This study aimed to use samples from unaffected chickens to examine the spatial and sex effects on the occurrence of WBD. We used samples from unaffected birds because we aim to study the innate biological differences between sexes of birds and regions of the P. major muscle without any discernible clinical, gross and histological changes attributable to WBD. There are a few important reasons we chose to study birds at 3 weeks of age. At this point in their life, broiler chickens do not typically present discernible clinical or gross changes associated with WBD. Detection of these changes frequently occur after 3 weeks of age (Papah et al., 2017). Hence, by utilizing 3-week-old birds, we are simultaneously selecting birds that do not have apparent clinical disease, but may be exhibiting molecular signatures of the developing disease that would become fulminant later in life. Indeed, a recent study in our laboratory demonstrated that molecular changes associated with WBD precede its clinical/gross presentation (Papah et al., 2018). Another important factor is that at 3 weeks of age, male and female broilers are beginning to diverge in terms of body weight and growth rates, with males growing faster and larger. Up until this point, male and female broilers grow similarly. Results from the current study supports this observation (see Figure 1). By studying birds that are at this point of divergence, we have a higher chance of seeing differences in gene expression between the sexes.

To identify and determine samples to be used in this study, all muscle tissue samples were subjected to microscopic analysis for tissues changes due to WBD; this was in addition to gross evaluation. Processing of samples for histologic assessment followed routine H/E protocol as used in the previous study (Papah et al., 2017) where cross and longitudinal sections of the tissues were obtained.

**FIGURE 2:**
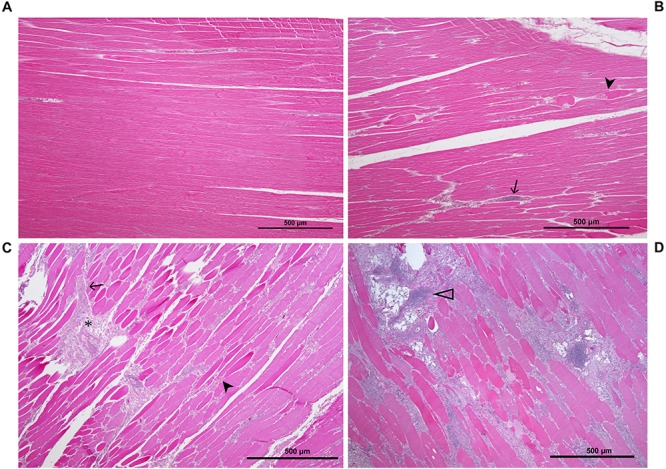
Histological sections of P. major sample showing the various stages of pathology associated with WB: **(A)** unaffected muscle sample; **(B)** mildly affected muscle tissue exhibiting multifocal myodegeneration (arrowhead) and focal infiltration by inflammatory cells (arrow); **(C)** moderately affected sample as shown by diffuse myofiber degeneration multifocal inflammatory cell infiltration and fibrosis (asterisk); **(D)** severely/markedly affected muscle sample as indicated by diffuse myodegeneration, diffuse myofiber inflammation, lipid infiltration and phlebitis (open arrowhead).

To classify all 24 samples on the basis of degree of tissue pathology associated with WBD, four categories were identified; namely unaffected, mild, moderate, and severe (Table 1). The degree and extent of microscopic lesions in the two sections (cross and longitudinal) per sample was used to determine the degree of tissue pathology under the four categories. Microscopic parameters used included myofiber degeneration and fragmentation, inflammatory cell infiltration, interstitial edema, necrosis, variability in myofiber sizes, lipid infiltration and fibrosis. Samples without any lesions or subtle focal degeneration (occupying <5% of the tissue slide) were placed under the unaffected category. Samples exhibiting single-cell myodegenerative changes occupying 5–25% of the tissue slide with focal infiltration of inflammatory cells were placed under the mild category. Samples with 25–50% of the tissue slide displaying myodegenerative changes with multifocal inflammation and focal interstitial edema were considered to be moderate. Samples with multifocal to diffuse myodegeneration over >50% of the tissue slide, myonecrosis and inflammatory cell infiltration as well as variability of fiber sizes and focal lipid infiltration were considered to be severely affected (Figure 2). It should be noted that muscle samples from the cranial and caudal regions were examined independently. Therefore, for a sample to qualify for RNA-sequencing analysis in the current study, both cranial and caudal samples had to be unaffected.

**TABLE 1:** Histological presentation of pectoral muscle samples harvested from the cranial and caudal pectoral regions of broiler chickens at 3 weeks of age.

**Cranial pectoral**	**Caudal pectoral**	**Number of samples**
Normal	Normal	7
Mild	Normal	5
Mild	Mild	5
Mild	Moderate	2
Moderate	Normal	2
Moderate	Mild	1
Moderate	Moderate	1
Severe	Moderate	1
Total		24

Pectoralis major muscle samples from 7 birds exhibited normal morphology in both the cranial and caudal portions, and 12 samples from 6 birds (3 males and 3 females) were selected for RNA-sequencing (Table 2). We performed Principal Component Analysis to ensure that our sample size was adequate for the study (Supplementary Figure 1).

**TABLE 2:** Total body masses of birds used in the RNA-seq study.

**Bird ID#**	**Sex**	**Total body mass (g)**
484951	M	1068
485058	M	982
485061	M	1000
484918	F	888
485060	F	846
485067	F	869

### RNA Sequencing

A total of 12 cranial and caudal tissue samples from male and female birds were used for RNA extraction and sequencing. RNA was isolated from the samples using the mirVana miRNA Isolation Kit (Thermo Fisher Scientific). RNA quality was tested using Nanodrop 1000 and Fragment Analyzer, which confirmed that the quality of the isolated RNA was acceptable for it to be used throughout the rest of the protocol. Complementary DNA (cDNA) libraries were synthesized using the TruSeq Stranded mRNA Sample Preparation Kit (Illumina), utilizing low sample protocol. Libraries were then sent to the Delaware Biotechnology Institute (DBI) where they were paired-end (2X101) sequenced with Illumina HiSeq 2500.

### Data Analysis

The quality of the resulting sequences was checked using FastQC v0.11.5. Low quality bases were trimmed with Trimmomatic v0.36, using parameters TRAILING:20 and SLIDINGWINDOW:4:15. After trimming, the reads were aligned to the chicken reference genome Gallus_gallus-5.0.89 using HISAT v2.1.0, then differential expression analysis was performed using Cuffdiff v2.2.1. Significant genes were defined as those with a false discovery rate (FDR) adjusted *p*-value of less than 0.05. Differential expression analysis of cranial vs. caudal aspects of the P. major muscle was performed separately for males and females. The analyses detected no DEGs in females and only 12 in males, indicating a nearly consistent gene expression profile between cranial and caudal aspects of the muscle in both male and female datasets. For greater statistical power, cranial and caudal data were combined for males vs. female analysis, and likewise male and female data were combined for cranial vs. caudal comparison. To identify biological processes and pathways that are significantly enriched by the differentially expressed genes, the gene list was analyzed using the Database for Annotation, Visualization and Integrated Discovery (DAVID) v6.8 (Huang et al., 2009a, b) and Ingenuity Pathways Analysis (IPA) by QIAGEN (Krämer et al., 2014).

## Results

### Body Weight Analysis

A linear regression analysis was performed using JMP statistical software (JMP Pro^®^ Version 13, SAS Institute Inc.) to determine statistical significance of body weight differences between male and female broilers over time, and to confirm the divergence of body weight and growth rate between male and female broilers at 3 weeks of age (Figure 1). Except for the first day of life, body weight differed significantly (*p*-value ≤ 0.0001) between males and females in all days of weight measurement (Supplementary Table 1).

### RNA-Seq Analysis

#### Cranial vs. Caudal

There were 12 differentially expressed genes found between cranial and caudal tissue samples (Table 3). Eight of these genes were upregulated and four were downregulated in the cranial samples.

**TABLE 3:** Cranial vs. caudal differentially expressed genes.

**Gene**	**Caudal FPKM**	**Cranial FPKM**	**Log2 (fold change)^1^**
PITX2	1.44	4.47	1.63
HOXA11	1.05	3.20	1.60
NRN1	2.81	7.93	1.50
PLIN1	3.98	9.52	1.26
FABP4	47.22	91.08	0.95
HBAD	37.58	70.28	0.90
KY	26.75	49.26	0.88
G0S2	49.17	90.03	0.87
FMOD	21.15	8.65	–1.29
COL12A1	4.76	1.59	–1.59
COL22A1	1.52	0.45	–1.76
COL11A1	6.14	1.71	–1.88

*^1^A positive (or negative) log2 (fold change) signifies higher (or lower) expression of the corresponding gene in the cranial aspect of the muscle.*

As there were only 12 significant DEGs in the cranial versus caudal comparison, IPA and DAVID analysis were not performed on this dataset. Rather, we explored the functions of these genes individually: Perilipin 1 (PLIN1), G0/G1 switch gene 2 (G0S2), and fatty acid-binding protein 4 (FABP4) are all involved in lipid metabolism; neuritin 1 (NRN1) is involved in nervous system development; kyphoscoliosis peptidase (KY) is involved in muscle growth, and homeobox A11 (HOXA11) and pituitary homeobox 2 (PITX2) are transcription factors. HBAD refers to the gene coding for hemoglobin subunit alpha-D. Fibromodulin (FMOD), collagen type XI alpha 1 chain (COL11A1), collagen type XII alpha 1 chain (COL12A1), and collagen type XXII alpha 1 chain (COL22A1) are all involved in collagen synthesis and interactions (Gene, 2004).

#### Male vs. Female

A total of 260 genes were differentially expressed between males and females, 189 of which were upregulated in males. Of the 71 genes downregulated in males, 13 of them had expression values of or very close to zero. These genes were mapped to the W chromosome (3 genes) or unplaced scaffolds (9 genes), except for one gene mapped to the Z chromosome, which is likely a genome assembly error. Because they demonstrate an FPKM value of 1.7–43.4 in females but zero in males, these genes on unplaced scaffolds are also likely to be on the W chromosome, which does not exist in male birds. The top 10 upregulated and downregulated genes in males are identified in Table 4.

**TABLE 4:** Top 10 upregulated and downregulated genes in males^1^.

**Gene**	**Female FPKM**	**Male FPKM**	**Log2 (Fold Change)**
CHAC1	1.53	14.28	3.22
ENSGALG00000046114	0.85	7.23	3.09
C7	1.79	8.10	2.18
PDK4	1.96	8.71	2.16
NOV	0.41	1.79	2.12
RPL3L	1.69	6.98	2.04
CCK	3.22	12.08	1.91
FMOD	6.57	21.76	1.73
CSMD1	1.07	3.46	1.69
MT4	42.37	133.88	1.66
CD24	4.94	1.62	–1.60
TNNI1	22.05	6.96	–1.66
CLEC19A	4.09	1.29	–1.67
GCH1	4.95	1.33	–1.90
ENSGALG00000044018	3.19	0.83	–1.94
AMPH	9.26	2.25	–2.04
ENSGALG00000036306	3.05	0.56	–2.45
RAPGEF4	3.51	0.46	–2.93
KCHIP2	2.14	0.27	–2.96
ENSGALG00000029783	4.22	0.11	–5.22

*^1^Excludes 13 genes with male FPKM values of less than 0.1, located on the W chromosome or an unplaced scaffold. A positive (or negative) log2 (fold change) signifies higher (or lower) expression of the corresponding gene in male birds.*

Of the 260 differentially expressed genes between males and females of the current study, 58 overlapped with differentially expressed genes found in the previous study by Papah et al. (2018) studying differential gene expression between WBD-affected and unaffected 3-week-old male broilers (Supplementary Figure 2). Of the 58 overlapping genes, 47 of them (81%) shared the same directionality.

Ingenuity Pathway Analysis of the differentially expressed genes between male and female unaffected samples provided predictions of differentially regulated biological functions (Table 5) and pathways (Table 6), as well as predicting the possible activation or deactivation of upstream regulators (Table 7).

**TABLE 5:** Top biological functions for males vs. females.

**Biological function**	***P*-value**	**Number of molecules**
**Diseases and disorders**		
Cancer	7.86E-03 – 2.39E-08	163
Organismal injury and	7.86E-03 – 2.39E-08	165
Abnormalities		
Cardiovascular disease	7.86E-03 – 4.77E-07	43
Neurological disease	7.86E-03 – 4.77E-07	41
Gastrointestinal disease	7.73E-03 – 1.19E-06	154
**Molecular and cellular functions**		
Cell morphology	7.86E-03 – 1.37E-06	37
Lipid metabolism	7.86E-03 – 2.90E-06	31
Small molecule biochemistry	7.86E-03 – 2.90E-06	44
Molecular transport	7.73E-03 – 2.94E-06	26
Cell death and survival	7.86E-03 – 4.40E-06	64
**Physiological system development and function**		
Cardiovascular system	7.86E-03 – 1.58E-07	43
Development and function		
Organ development	7.66E-03 – 1.58E-07	32
Organ morphology	7.86E-03 – 1.35E-06	43
Skeletal and muscular system	7.86E-03 – 1.37E-06	43
Development and function		
Tissue morphology	7.73E-03 – 1.37E-06	54

**TABLE 6:** Top ten canonical pathways for males vs. females.

**Canonical pathway**	***P*-value**	**Molecules**
D-myo-inositol (1,4,5,6)-	9.85E-04	6
tetrakisphosphate biosynthesis		
D-myo-inositol (3,4,5,6)-	9.85E-04	6
tetrakisphosphate biosynthesis		
3-Phosphoinositide	1.05E-03	7
Biosynthesis		
3-Phosphoinositide	1.59E-03	6
Degradation		
D-myo-inositol-5-phosphate	1.80E-03	6
Metabolism		
Fatty acid β-oxidation I	2.00E-03	3
superpathway of inositol	2.63E-03	7
phosphate compounds		
complement system	3.05E-03	3
retinol biosynthesis	4.38E-03	3
IGF-1 signaling	9.84E-03	4

**TABLE 7:** Significant activated and inhibited upstream regulators.

**Upstream regulator**	**Molecule type**	**Activation Z-score**	**Overlapping *P*-value**
ACOX1	Enzyme	–2.45	1.21E-03
MYOD1	Transcription regulator	–2.41	4.70E-03
N-cor	Group	–2.22	9.72E-05
CD44	Other	2.00	6.62E-03
MAPK9	Kinase	2.00	3.72E-02
CTNNB1	Transcription regulator	2.00	2.32E-02
IL6	Cytokine	2.07	3.72E-02
ERK	Group	2.20	3.61E-03
NCOA2	Transcription regulator	2.22	3.77E-04
TGFB3	Growth factor	2.24	1.96E-03
CEBPA	Transcription regulator	2.35	3.58E-03
FOXO1	Transcription regulator	2.42	5.54E-05
PPARA	Ligand-dependent nuclear receptor	2.54	2.41E-05
NFE2L2	Transcription regulator	2.58	2.32E-03

Additionally, by entering a list of the genes upregulated in males into DAVID we were able to find gene ontologies upregulated in male birds. DAVID analysis yielded 12 main gene ontologies upregulated in male broilers (Table 8). “Gene Count” refers to the number of genes associated with a given ontology, out of the 88 gene IDs DAVID accepted from the list of 189 genes upregulated in male broilers. Entering a separate list of the 71 downregulated genes in males did not yield significant results from DAVID.

**TABLE 8:** DAVID ontologies for genes expressed higher in male.

**Ontology**	**Gene count**	***P*-value**
Coagulation	7	4.41E-04
Cytokine production	8	6.99E-04
Response to wounding	9	1.76E-03
Cell adhesion	10	2.37E-03
Foam cell differentiation	3	3.19E-03
Lipid metabolism	4	3.23E-03
Protein metabolism	4	3.89E-03
Antiangiogenesis	4	7.65E-03
Extracellular matrix organization	6	8.66E-03
Tissue development	17	1.34E-02
Immune response	12	1.35E-02
Oxidative stress	6	2.27E-02

## Discussion

### Fat Metabolism

Analysis of differentially expressed genes between regions of the pectoralis major muscle and sexes of birds strongly suggests increased fat metabolism and deposition in male birds and the cranial aspect of the breast muscle. This correlates with the increased fat content found in the P. major muscle of birds affected with Wooden Breast and/or white striping (Mudalal et al., 2014; Soglia et al., 2016) as well as evidence of fatty infiltration observed in histological studies even before wooden breast is clinically and grossly detectable (Papah et al., 2017). This suggests that the breast muscle in male broilers, as well as the cranial aspect of the breast muscle, have a higher tendency to accumulate lipids, which may make them more susceptible to WBD.

For example, fatty acid-binding protein 4 (FABP4), which was upregulated in males, plays a major role in fat accumulation by acting as a fatty acid carrier and segregating fatty acids for triglyceride synthesis to develop adipose tissue (Smathers and Petersen, 2011). This protein is also a biomarker for adipocyte differentiation (Zhang et al., 2015). Similarly, lipoprotein lipase (LPL), upregulated in male birds, is an effective biomarker for adipogenesis. LPL, whose active form is found on the endothelium of capillaries and small-caliber blood vessels, acts by hydrolyzing triglycerides in blood into free fatty acids for oxidative phosphorylation by other cells. Additionally, the free fatty acids can be re-esterified into triglycerides for storage in adipocytes (Zhang et al., 2015). This finding is in line with a recent study in our laboratory that showed upregulation of FABP4 in the P. major muscles of WBD-affected chickens at week 3 of age (Papah et al., 2018). Taken together, these studies suggest that active intracellular mobilization of lipids accompanies the early phase of WBD in chickens.

Fatty acid translocase (CD36), upregulated in males, is a receptor for multiple types of ligands, including long-chain fatty acids. It behaves as a fatty acid translocase in adipose tissue, as well as cardiac and skeletal muscle (Febbraio et al., 2001), and therefore may play a role in fat accumulation. In agreement, palmitic acid was predicted by IPA as an activated upstream regulator in males, suggesting higher levels of this long chain fatty acid in the breast muscle of males compared with females. CD36 has been found to play a role in other pathways that are significant to WBD, included antiangiogenesis and immune functions (Febbraio et al., 2001).

**FIGURE 3:**
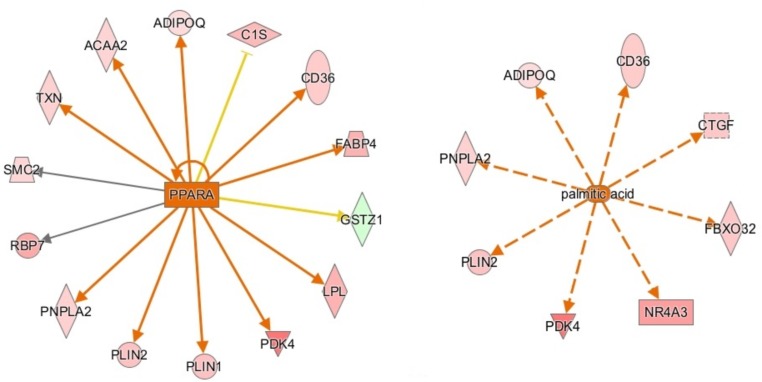
Activation network diagrams for PPARA (left) and palmitic acid (right). Pink and green shapes represent genes upregulated or downregulated in males, respectively. Orange arrows represent IPA’s prediction that the upstream regulator activates the corresponding genes. Yellow arrows represent disagreement between the direction of the gene expression (i.e., up or downregulation) and IPA’s prediction of the relationship between the upstream regulator and the corresponding gene. Gray arrows mean that IPA predicts a relationship between the upstream regulator and the corresponding gene but cannot predict the exact effect. Image produced using IPA.

Ingenuity pathway analysis suggests activation of an upstream regulator that stimulates genes involved in fat metabolism. Peroxisome proliferator-activated receptor alpha (PPARA) is a ligand-dependent nuclear receptor that regulates expression of multiple genes involved in lipid metabolism, including adiponectin (ADIPOQ), FABP4, CD36, perilipin 1 and 2 (PLIN1/PLIN2), and LPL. Because these genes and a few other PPARA target genes are upregulated in the male vs. female dataset, IPA predicted that PPARA is activated in male broilers. PPARA also plays key roles in regulating multiple steps of the fatty acid β-oxidation (Lemberger et al., 1996), supporting a hypothesis of increased mitochondrial fatty acid β-oxidation in male modern broilers that will be discussed further in the next section. IPA activation networks for PPARA and palmitic acid are shown in Figure 3.

Additional significant genes involved in fat deposition and metabolism include patatin-like phospholipase domain-containing protein 2 (PNPLA2), pyruvate dehydrogenase kinase 4 (PDK4), retinol binding protein 7 (RBP7), solute carrier family 44 member 1 (SLC44A1), and acetyl-CoA acyltransferase 2 (ACAA2), all upregulated in the P. major muscle of males. ACAA2 is a gene with key involvement in mitochondrial fatty acid β-oxidation. It catalyzes the final step in the β-oxidation pathway, in which β-ketoacyl-CoA reacts with a free molecule of coenzyme A to produce acetyl-CoA (Nelson and Cox, 2008).

It has been observed that abdominal fat is the most variable body component of broiler chickens, and that female broilers tend to have more abdominal cavity fat deposition than males (Leenstra, 1986), which is consistent with the lower feed efficiency of females. It is well known that increased abdominal fat deposition is associated with the lower feed efficiency in chickens (Zhuo et al., 2015). These findings from prior studies agree with our results that suggests male broilers have higher uptake and oxidation of lipids by the P. major muscle. It can be hypothesized that female broilers catabolize lipids at a lower rate than male broilers due to lower expression of fat metabolism genes in the muscle, and as a result they have more accumulation of fat in the abdominal cavity.

### Metabolic Shift

Evidence of increased fat metabolism in the pectoralis major muscle of male birds is especially significant because it opposes the normal metabolism of the muscle. The pectoralis major in chickens is a “white” muscle, meaning it consists almost entirely of type II fast-twitch fibers (Ono et al., 1993; Verdiglione and Cassandro, 2013), contains little myoglobin (hence the “white” color) (Kranen et al., 1999), operates primarily on glycolytic metabolism, and performs little to no oxidative phosphorylation of fats (Remignon et al., 1994). Remignon et al. (1994) found that two key enzymes involved in fatty acid oxidation, citrate synthase and 3-hydroxyl-CoA dehydrogenase, had significantly lower activity than lactate dehydrogenase, a key enzyme in glycolysis, in the P. major of broiler chickens. These observations held true for both fast- and slow-growing broiler lines. Because oxidative metabolism normally contributes little energy to the chicken breast muscle compared to glycolytic metabolism, it is unusual that we observed increased expression of fat metabolism-related genes in the muscle.

This data suggests a metabolic shift from primarily glycolytic metabolism to increasing proportions of fatty acid oxidative metabolism. An important gene supporting this hypothesis is LPL, which was found to decrease glucose metabolism when overexpressed in mice (Voshol et al., 2001). Evidence of increased fatty acid oxidation and redirection of carbohydrates from glycolysis to other metabolic pathways such as the pentose phosphate, glucuronic and hexosamine biosynthetic pathways have been noted in birds affected by WBD (Abasht et al., 2016; Papah et al., 2018) and also in high feed-efficiency broilers which are more susceptible to WBD (Abasht et al., 2019). Therefore decreased glycolytic activities and increased uptake and oxidation of fatty acids appear to be a key feature of WBD.

Palamiuc et al. (2015) described inhibition of glucose metabolism and a shift of fuel preference to lipids during a metabolic shift event early in the development of amyotrophic lateral sclerosis (ALS) in mice. Affected mice experienced increased expression of LPL, CD36, PDK4, forkhead box O1 (FOXO1), and PPARβ/γ during this metabolic shift. LPL, CD36, and PDK4 are upregulated in males in our data set. FOXO1 and PPARβ/γ are not upregulated in males in our dataset, but FOXO1 was predicted to be an activated upstream regulator by IPA. Whether increased expression of one of these genes, increased fatty acid concentration in the muscle, or another factor entirely outside of this pathway is the initial cause of the hypothesized metabolic shift in WBD has yet to be deciphered.

It is likely that the shift from glycolytic to oxidative metabolism in the muscle is a change that is energetically beneficial for faster growth and higher feed efficiency, but has had detrimental effects on the breast muscles of modern broilers. Type II muscle fibers are poor at scavenging H_2_O_2_, allowing accumulation of reactive oxygen species (ROS) in the muscle when lipids are utilized for extended periods of time, which is known to lead to mitochondrial dysfunction and muscular damage (Anderson and Neufer, 2006). This hypothesis is in agreement with a study by Papah et al. (2018) in which WBD-affected broilers demonstrated increased mitochondrial dysfunction. Affected mice that underwent the metabolic shift in Palamiuc et al., 2015 study displayed evidence of ROS accumulation. Mechanisms of oxidative stress and its effect on the chicken breast muscle are further discussed in Section “Oxidative Stress.”

### Oxidative Stress

Oxidative stress is the accumulation of ROS generated through metabolic reactions, and the resulting imbalance between ROS and antioxidant substances, which can lead to damage of DNA (Cooke et al., 2003) and injury of tissues due to reactions involving ROS, such as lipid peroxidation and protein damage (Betteridge, 2000). IPA and DAVID analysis predict that biological reactions to oxidative stress are upregulated in male birds. The reason for this could be that modern male broilers grow faster and larger than their female counterparts, and are therefore generating ROS at a higher rate, but do not have sufficient mechanisms to counteract ROS accumulation. Male broilers’ increased susceptibility to oxidative stress may play a large part in their increased susceptibility to WBD, and supports a hypothesis that oxidative stress plays a role in the development of WBD.

The aforementioned RNA-sequencing study by Mutryn et al. (2015) identified oxidative stress as a potential factor in the development of WBD when they found many DEGs associated with an increased ROS in WBD-affected birds. Additionally, Abasht et al. (2016) found that WBD-affected birds exhibited increased biomarkers related to oxidative stress, as well as increased levels of molecules that could affect redox homeostasis, including xanthine, hypoxanthine, and urate. In the current study, upregulated genes involved in oxidative stress responses include aprataxin (APTX), NR4A3, DNA damage recognition and repair factor (XPA), TXN, ADIPOQ, CD36, POSTN, oxidative stress-induced growth inhibitor 1 (OSGIN1), G protein subunit alpha Q (GNAQ), RET, cytokine inducible SH2-containing protein (CISH), ChaC glutathione specific gamma-glutamylcyclotransferase 1 (CHAC1), and cholecystokinin (CCK). Some of these genes are involved in attempt to relieve and protect against the damage of oxidative stress. For example, it has been discovered that adiponectin, coded for by ADIPOQ, protects cells against cytotoxicity when under oxidative stress by activating adenosine monophosphate-activated protein kinase (AMPK), which in turn increases metabolism of glucose and fatty acids (Chan et al., 2012). Additionally, TXN is a regulator of cellular responses to oxidative stress, and overexpression of TXN protects against cytotoxicity resulting from oxidative stress (Nishinaka et al., 2013).

However, the higher expression of CHAC1, which was the top upregulated gene in male broilers, may have a detrimental effect to the muscle. A prior study found that CHAC1 expression is increased by stress in the endoplasmic reticulum, and its protein degrades glutathione in the cell (Crawford et al., 2015). This is important because glutathione is a major antioxidant in cells, and its depletion results in oxidative stress (Crawford et al., 2015). The higher expression of CHAC1 in male broilers suggests that glutathione is being degraded at a higher rate than normal, which may be playing a big role in oxidative stress in male broilers. CHAC1 is also a member of the unfolded protein response (UPR) pathway and promotes apoptosis (Mungrue et al., 2009). However, because our data doesn’t show differential expression of other key genes in the UPR pathway, such as CHOP, PERK, XBP, and ATF1, we can assume that the UPR pathway isn’t regulated differently between sexes of birds.

**FIGURE 4:**
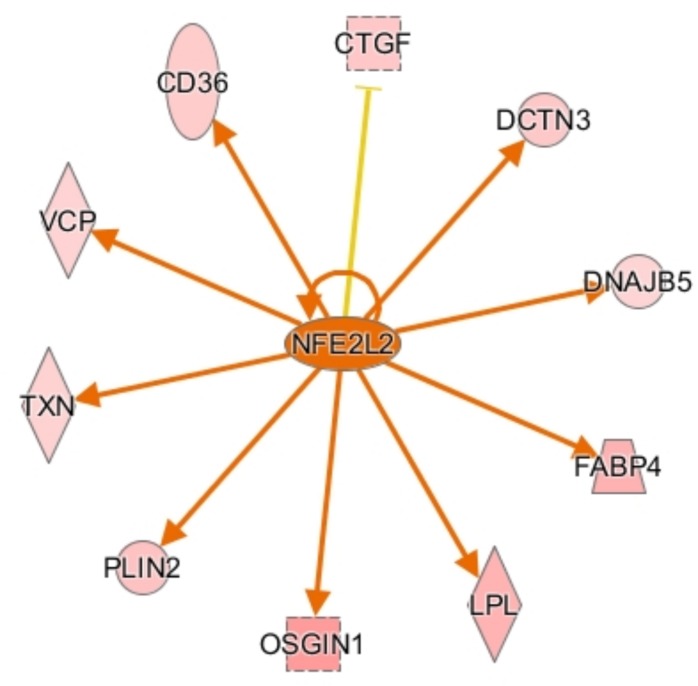
Activation network diagram for NFE2L2. Pink shapes represent genes that were upregulated in the dataset. Orange arrows demonstrate that IPA predicts NFE2L2 activates the corresponding genes. The yellow line to CTGF means that the fold change for CTGF is not consistent with IPA’s predicted relationship between NFE2L2 and CTGF. Image produced using IPA.

CCAAT enhancer binding protein α (CEBPA) is a transcription factor involved in stimulating oxidative stress responses from other genes downstream, and it was predicted to be activated in males by IPA. CEBPA and other CEBP-family proteins undergo increased expression under stress, and in turn they increase expression of other oxidative stress response genes (Huggins et al., 2016), including ADIPOQ and TXN. Another activated transcription regulator, NFE2L2, acts similarly by mediating stress and inflammatory responses through regulation of CD36, OSGIN1, DNAJ heat shock protein family member B5 (DNAJB5), and ANXA1 (Figure 4). Upregulation of these stress response genes suggests the existence of increased oxidative stress in male birds, which could be a key factor in their susceptibility to WBD.

### Vascular Damage

DAVID and IPA suggest that blood clotting and antiangiogenic functions are upregulated in male broilers. Additionally, IPA predicts increased cardiovascular damage in males, with significant related diseases and disorders including atherosclerosis, vascular lesions, and vaso-occlusion. These results are in agreement with a study by Papah et al. (2018). The results suggest that compared with females, male birds are more susceptible to vascular damage and increased antiangiogenic activity, even without being affected by any degree of WBD. This correlates with the observed presence of inflammation and lipogranulomas in the vasculature of WBD-affected birds (Papah et al., 2017), as well as a hypothesis that localized hypoxia caused by increased oxygen demand and compromised vasculature may be a factor in the development of WBD (Mutryn et al., 2015).

Like vascular damage, inhibition of vasculogenesis can also lead to inadequate delivery of oxygen and nutrients to skeletal muscle, and could be a factor in the development of WBD (Sihvo et al., 2018). Male broilers, which grow more quickly than females, would require a concomitant increase in angiogenic activity to ensure that their rapidly-growing muscle tissue has proper vasculature. Though there were some angiogenic genes upregulated in males, such as annexin A-1 (ANXA1) and endoplasmic reticulum aminopeptidase 1 (ERAP1), they appear to be overtaken by multiple upregulated antiangiogenic genes. For example, thrombospondin 1 and 2 (THBS1 and THBS2) are known antiangiogenic genes upregulated in male broilers. They inhibit angiogenesis by interference with endothelial cell migration, as well as competing with growth factors to bind to proteoglycans on endothelial cell surfaces (Tolsma et al., 1993; Lawler, 2000).

THBS1, as well as CD36, ADIPOQ, LPL, serpine family E member 2 (SERPINE2), coagulation factor XIII A chain (F13A1), and G protein subunit Alpha Q (GNAQ) are all upregulated and involved in blood coagulation and clotting, both of which were found to be upregulated functions in male broilers in DAVID and IPA. This makes sense, as it is known that the activation of coagulation is largely mediated by inflammatory cytokines (Esmon, 2005). Interestingly, a study by Mutryn et al. (2015) showed that coagulation pathways were downregulated in WBD-affected birds, despite inflammatory pathways being upregulated. Perhaps this is due to natural anti-coagulant mechanisms kicking in to compensate for persisting coagulation and inflammation in later stages of WBD to prevent further damage to the body. The involvement of coagulation mechanisms in broiler growth and WBD require further investigation.

### Connective Tissue Proliferation

DAVID and IPA both predict that proliferation of connective tissue is being significantly upregulated in male birds. A male predisposition for connective tissue growth correlates with findings of fibrosis and increased collagen levels in histological studies (Papah et al., 2017) as well as gene expression of WBD-affected tissues (Mutryn et al., 2015; Papah et al., 2018), which may be a factor of the stiffness of WBD-affected muscle (Mudalal et al., 2014; Soglia et al., 2016). Studies confirm increased collagen content throughout the bodies of male chickens, including the P. major muscle (Granot et al., 1991; Sakakibara et al., 2010). Males’ increased propensity for connective tissue proliferation may be an important factor in their increased susceptibility to WBD.

As an example, connective tissue growth factor (CTGF), upregulated in males, is highly fibrogenic. It stimulates connective tissue proliferation and extracellular matrix (ECM) development, and is overexpressed in many fibrotic lesions (Rachfal and Brigstock, 2003). Additionally, CTGF has been identified as being transcriptionally regulated by transforming growth factor-beta (TGF-β) (Rachfal and Brigstock, 2003), which was also predicted by IPA to be an activated upstream regulator in male broilers. Transforming growth factor beta-induced (TGFBI) is upregulated in males as well, and plays key roles in ECM development and ECM-muscle cytoskeleton interactions in mice and humans, as well as myofiber growth and myofibril bundling in zebrafish embryos (Kim and Ingham, 2009).

Additionally, periostin (POSTN) was upregulated in males. POSTN is most well-known for its involvement in osteogenesis but is also expressed in collagen-rich connective tissues throughout the body—mostly those that are subjected to constant mechanical stress (Norris et al., 2007). Considering the physical stress put on the P. major during its rapid growth in modern broilers, it is reasonable to hypothesize that the increased expression of POSTN in males could contribute to higher collagen content in male broiler breast muscles, as well as increasing their susceptibility to WBD. This hypothesis may be supported by upregulation of small muscle protein x-linked (SMPX) and nuclear receptor subfamily 4 group A member 3 (NR4A3) in male broilers. SMPX is a gene regulated downstream of NR4A3 and is involved in muscle regulation. The exact mechanism of SMPX isn’t currently clear, as there are conflicting studies arguing whether or not it is involved in muscle growth and differentiation (Eftestøl et al., 2014; Ferrán et al., 2016). However, there are studies suggesting that SMPX may be related to mechanical stress (Eftestøl et al., 2014), supporting the idea that increased biomechanical stress in the breast muscle may be a contributing factor to differential expression of genes between regions of the muscle and sexes of birds, as well as contributing to the development of WBD.

Additional DEGs (males vs. females) indicated as being involved in connective tissue proliferation by IPA include tenomodulin (TNMD), ret proto-oncogene (RET), histidine triad nucleotide binding protein 1 (HINT1), x-ray repair cross-complementing 4 (XRCC4), RAS P21 protein activator 1 (RASA1), thioredoxin (TXN), cyclin D3 (CCND3), nephroblastoma-overexpressed gene (NOV), ADIPOQ, fibulin 1 (FBLN1), Dickkopf WNT signaling pathway inhibitor 3 (DKK3), and klotho (KL).

### Muscle Development

As previously explained, a key predisposing factor of WBD is increased muscle growth rates and yields in modern broiler chickens. By examining genes involved in muscle growth we seek to find differences in expression that could explain the cause of increased growth rates and muscle yields in male broilers and the cranial aspect of the breast muscle.

Unexpectedly, F-box 32 (FBXO32) was upregulated in male broilers. This is unusual because FBXO32 is involved in muscle atrophy by behaving as a ubiquitin protein ligase and enhancing proteolysis (Gomes et al., 2001). Increased expression of FBXO32 could be a response to muscle damage caused by oxidative stress, or by hypoxia as hypothesized by Mutryn et al. (2015).

While there are some DEGs involved in muscle growth in cranial vs. caudal and male vs. female analysis, there are many genes with key roles in growth that don’t exhibit differential expression in either analysis, such as insulin-like growth factors 1 and 2 (IGF-1 and IGF-2), fibroblast growth factor (FGF), hepatocyte growth factor (HGF), platelet-derive growth factor (PDGF), myostatin (MSTN), myogenin (MYOG), and myoferlin (MYOF) (McFarland, 1999). This makes it difficult to draw any conclusions on the involvement of differential gene expression at week 3 post-hatch in increased growth in male broilers and the cranial aspect of the breast muscle.

Rather than differential gene expression at 3 weeks of age mediating increased growth in male birds and the cranial region of the muscle, it is possible that differences in muscle growth are caused by the morphology of the muscle fibers themselves, decided before 3 weeks of age. Smith and Fletcher (1987) found that myofibers in the cranial region of the pectoralis major muscle had significantly smaller cross-sectional areas than those in the caudal region, suggesting a higher myofiber density in the cranial region. Additionally, studies also report a higher myofiber density and number in the muscles of male chickens both pre- and post-hatch, as well as reporting as smaller average cross-sectional area in male myofibers than in females (Henry and Burke, 1998; Scheuermann et al., 2003). The larger number of myofibers in modern male broiler chickens and the cranial aspect of the breast muscle could contribute to their increased growth rate and mass.

### Z Chromosome

Out of the 189 genes upregulated in the pectoralis major of male broilers, 103 are located on the Z chromosome. Because we aimed to uncover the biological basis of increased growth rates in male broiler chickens, differential expression of genes on the sex chromosomes is of particular interest to us. In birds, the sex chromosomes are “W” and “Z,” with females being heterogametic (ZW) and males homogametic (ZZ).

**FIGURE 5:**
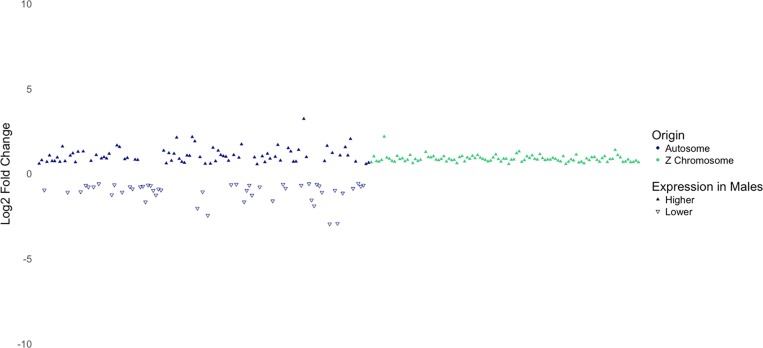
Log2 fold-change of differentially expressed genes separated by whether they are located on an autosome or the Z chromosome. Notice the consistency in log2-fold-change values of genes on the Z chromosome.

Dosage compensation is a mechanism that balances gene expression on sex chromosomes between males and females to compensate for differences in expression caused by differences in copy numbers of the sex chromosomes (Nguyen and Disteche, 2006). However, it has been found that the dosage compensation mechanism in birds is not as effective as it is in mammals. While the male-to-female ratio for expression of X-linked genes in mammals is typically close to 1.00 (Nguyen and Disteche, 2006), the male-to-female ratio for expression of Z-linked genes in birds has been reported to be between 1.4 and 1.6 by Ellegren et al. (2007), and between 1.33 and 1.58 by Itoh et al. (2007). In agreement to these findings, a recent study from our laboratory reported that genes on the Z chromosome in male broiler chickens are expressed 1.35 times higher than that in females (Zhuo et al., 2017). As shown in Figure 5, DEGs on the Z chromosome show more uniform log 2 fold-change values than DEGs on autosomes. In addition, except one gene (not shown in Figure 5), all DEGs on Z chromosome are expressed higher in males with a mean fold-change value of 1.84 (log 2 fold-change = 0.88). Collectively, these findings suggest that the copy number of the Z chromosome is likely to be responsible for higher expression of these genes in males.

The inherent upregulation of Z-linked genes in male birds may provide some insight on the cause of increased WBD susceptibility in male broilers. IPA provided a network of differentially expressed genes between males and females related to growth. However, very few of these genes could be related to body size or muscle growth. As an example, heat shock protein family B member 3 (HSPB3) is upregulated in males in our dataset. It is known to be highly expressed during myogenic differentiation and plays a role as a “quality control” factor (Sugiyama et al., 2000). Higher HSPB3 expression may reflect increased myogenic activity in male broilers, but it does not explain it.

There weren’t many upregulated Z-linked genes involved in muscle and body growth, but we found that there were several upregulated Z-linked genes in males that were related to fat metabolism and deposition, including PLIN2, LPL, ACAA2, ALC44A1, RPP25L, HINT1, and AP3B1. This suggests a possibility that male broilers innately perform more lipid metabolizing activities, which corresponds with the hypothesis that a metabolic shift toward increased lipid metabolism may play a role in WBD development.

It should be noted that this innate difference in fat metabolism between the sexes is most likely unrelated to hormonal differences. Rather, it is likely driven by cell-autonomous sex differences in gene expression. In birds, somatic cells have inherent sex identities, and different “sexes” of cells can respond differently to the same profile of gonadal hormones (Zhao et al., 2010). In addition to the avian cell-autonomous sex identity phenomenon, it has been implicated that sex hormone levels in birds may not contribute to juvenile growth or body weight differences between the sexes. A study by Burke and Edwards (1994) found that male turkeys castrated early in life did not demonstrate significantly different body weights at 3, 6, 9, 12, or 15 weeks of age from their uncastrated counterparts, suggesting that testosterone levels do not have an effect on the growth rate or body mass of poultry.

## Conclusion

Through RNA-sequencing and analysis using DAVID and IPA, we were able to identify differences in gene expression between male and female broiler chickens, as well as the cranial and caudal aspects of the P. major muscle, that lead us to identify potential factors involved in different susceptibility to WBD between sexes of birds and regions of the breast muscle. Upregulation of genes involved in fat metabolism and deposition, vascular lesions and antiangiogenesis, and oxidative stress responses in males give us insight into the predisposing factors that may make male broilers more susceptible to WBD. Predicted changes in the activation states of transcription regulators, upstream enzymes and kinases, etc., suggest that gene expression changes observed in the current study could be linked to alteration in the activity of a few upstream regulators. Importantly, differential expression of genes on the Z-chromosome may be due to a lack of complete dosage compensation.

Though there were few differentially expressed genes identified in the cranial versus caudal analysis, there is some upregulation of genes involved in fat metabolism and muscle development in the cranial aspect of the muscle. However, we are reluctant to conclude that this reflects the mechanism of increased growth in the cranial aspect, because many key genes involved in regulating muscle growth are not differentially expressed in cranial vs. caudal or male vs. female analysis.

Unaffected birds for the current study were selected through gross and histological evaluation; however, there is a possibility that the development of WBD has started in these samples at a molecular level, and hence, some of the findings in the current study may be relevant to the onset of molecular perturbations ultimately leading to cellular and tissue damage in WBD. In agreement with this conclusion, the data collected in this project correlates with data from previous analyses of WBD-affected birds (Mutryn et al., 2015), including early developmental stages of WBD (Papah et al., 2018). However, as suggested above, some of the findings in the current study, such as higher expression of genes on Z chromosome in males, may be relevant to fundamental biological sex differences in modern broilers. Future studies with a heritage line of broilers in which WBD is not known to occur may be of interest to verify whether such differences are also present in slow-growing lines of chickens.

## Data Availability

The RNA-seq data generated and analyzed for this study is deposited in NCBI’s Sequence Read Archive (Study Accession No. SRP198866 and Bioproject Accession No. PRJNA543674).

## Ethics Statement

Animal protocols for this study were approved by the University of Delaware Institutional Animal Care and Use Committee.

## Author Contributions

BA conceptualized and oversaw the study, contributed to the scientific discussion, and revised the manuscript. BB performed RNA isolation, contributed to preparation of cDNA libraries, conducted IPA and DAVID analysis, and wrote the original draft of the manuscript. MP conducted histopathological examination of muscle samples and drafted its relevant literature in the Section“Selection and Identification of Samples for RNA Sequencing.” ZZ contributed to preparation of the cDNA libraries and conducted bioinformatic (RNA-seq) analysis.

## Conflict of Interest Statement

The authors declare that the research was conducted in the absence of any commercial or financial relationships that could be construed as a potential conflict of interest.
