# Supplementary material for: RNA-Seq Analysis Reveals Spatial and Sex Differences in Pectoralis Major Muscle of Broiler Chickens Contributing to Difference in Susceptibility to Wooden Breast Disease
Source: Front Physiol. 2019 Jun 18;10:764. doi: 10.3389/fphys.2019.00764 (PMC6591452; doi:10.3389/fphys.2019.00764)
Supplement: Supplementary file 1 [file Data_Sheet_1.docx]

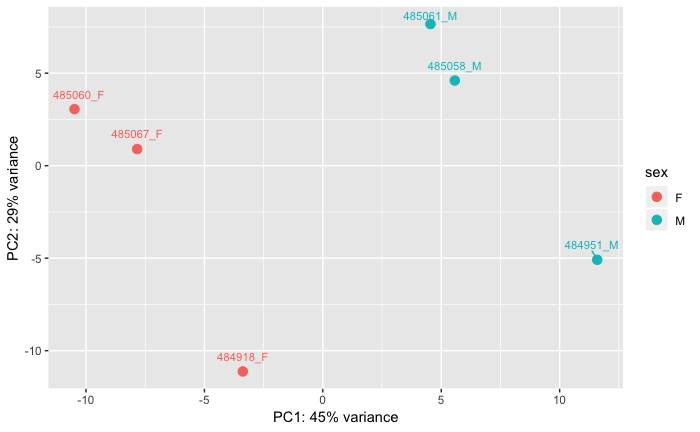


Supplementary Figure 1: Principal component analysis (PCA) of the normalized and regularized log-transformed count data. Raw reads counts were obtained using Stringtie (v1.3.5) and then normalized and regularized log-transformed using DESeq2 package (v1.22.2) in RStudio (vt.t.463). PCA plot was created using the plotPCA function from DEseq2 and ggplot2 (v3.1.0). (Wickham, 2016; Pertea et al., 105; RStudio Team, 2015; Love et al., 2014)

| Age (days) | Male Mean Weight (g) | Male Standard Error | Female Mean Weight (g) | Female Standard Error | P-Value |
| --- | --- | --- | --- | --- | --- |
| 1 | 47.74 | 0.45 | 46.73 | 0.40 | =0.10 |
| 7 | 158.67 | 1.80 | 149.18 | 1.63 | =0.0001 |
| 14 | 516.33 | 5.76 | 463.54 | 5.00 | <0.0001 |
| 21 | 1008.00 | 10.71 | 892.32 | 9.39 | <0.0001 |
| 28 | 1726.03 | 26.11 | 1527.29 | 21.41 | <0.0001 |
| 35 | 2605.66 | 31.89 | 2255.63 | 26.16 | <0.0001 |
| 42 | 3381.89 | 41.37 | 2894.00 | 33.93 | <0.0001 |
| 49 | 4180.56 | 43.86 | 3526.18 | 35.49 | <0.0001 |
| 56 | 4684.29 | 55.09 | 3953.21 | 44.77 | <0.0001 |

Supplementary Table 1: Broiler weight data by sex and age utilized to produce Figure 1 growth curve.


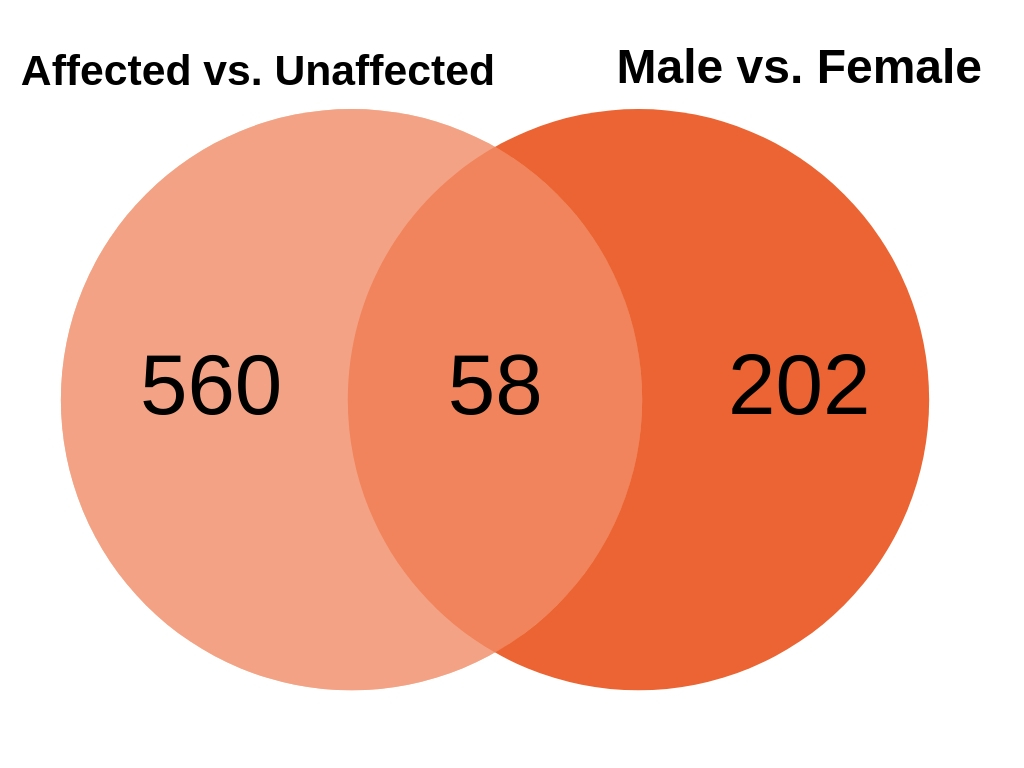


Supplementary Figure 2: Overlapping genes between the current study and a previous study from our lab studying gene expression between affected and unaffected birds at week 3 of life (Papah et al., 2018). Of the 58 overlapping genes between the dataset, 47 of them (81%) had the same directionality of expression.
